# Abnormalities in primary cilium of osteoblasts of adolescent idiopathic scoliosis patients

**DOI:** 10.1186/2046-2530-4-S1-P6

**Published:** 2015-07-13

**Authors:** N Oliazadeh, A Franco, D Wang, A Moreau

**Affiliations:** 1 Viscogliosi Laboratory in Molecular Genetics for Musculoskeletal Disease, Sainte-Justine University Hospital Research Center, Montreal, Québec, Canada; 2 Department of Biochemistry and Molecular Medicine, Faculty of Medicine, Montreal, Québec, Canada; 3 Department of Stomatology, Faculty of Dentistry, Université de Montréal, Montreal, Québec, Canada

## 

Adolescent Idiopathic Scoliosis (AIS) is a common complex genetic disease and one of the most prevalent childhood deformities. AIS is clinically characterized by a 3D spinal deformity with unknown cause. The aim of this study is to identify rare genetic variants and associated biological pathways involved in AIS onset and its progression.

We recently completed exome sequencing of 69 AIS cases vs. 70 matched controls in a French-Canadian AIS cohort using SOLiD 5 technology. Results showed two gene families with significant enrichment for single nucleotide variants (SNVs) involved in cilia signalling pathways. To investigate the functional effect of these SNVs, we explored biomechanical responses on osteoblasts from AIS patients carrying these SNVs. More specifically, we applied a sinusoidal/oscillatory fluid flow at 2Pa, 0.5Hz for 90min using parallel plate flow chamber.

Preliminary qPCR results of the cells post flow showed a significant increase (+10-folds) in the expression of cyclooxygenase-2 (COX2) in AIS cells versus control ones. Interestingly, microscopy imaging of the same cells revealed a 30% increase in the length of cilia (p<0.01) of AIS cells when compared to control ones. Of note, increased COX2 expression has been reported as a flow mediated response for bone cells. Our results suggest an increased sensitivity of AIS bone cells to fluid flow due to the elongation of cilia.

Although cilia abnormalities have never been investigated in idiopathic scoliosis, ciliary structural and functional abnormalities in AIS osteoblasts might be involved in the onset and progression of the disease. More extensive experiments are currently underway to confirm the role of cilia in AIS pathogenesis.

